# Safety of delaying endoscopic intervention for complete esophageal soft food bolus impaction

**DOI:** 10.1016/j.igie.2026.01.004

**Published:** 2026-01-27

**Authors:** Thomas Hoang, Andrew Fetz, Ranjit Andrew Singh

**Affiliations:** 1 Faculty of Medicine, Department of Medicine, University of British Columbia, Vancouver, British Columbia, Canada; 2 Faculty of Medicine, Division of Gastroenterology, Department of Medicine, University of British Columbia, Vancouver, British Columbia, Canada

## Abstract

**Background and Aims:**

Esophageal soft food bolus obstruction is a common emergency department presentation that requires urgent endoscopic intervention. Although current guidelines recommend intervention within 6 to 12 hours, recent evidence indicates that extending this timeframe has not been associated with worsening clinical outcomes. We aim to investigate the safety of delaying endoscopy beyond 6 hours of triage for complete esophageal food bolus obstructions.

**Methods:**

We conducted a multicenter retrospective cohort study evaluating coprimary outcomes of 30-day mortality and dysphagia-related hospitalizations and secondary outcomes of procedural adverse events among patients who underwent early (within 6 hours of triage) and delayed (after 6 hours of triage) endoscopic management of those with triage symptoms consistent with a complete esophageal food bolus impaction.

**Results:**

Mortality was not observed in either the early or delayed cohorts, and there was no difference in rehospitalization (4.4% vs 3.3%, respectively; *P* = 1). There was a numerical difference in mucosal injury (2.2% vs 13.2%) that was not statistically significant (*P* = .15). Similar rates of general anesthesia (6.67% vs 6.67%; *P* = 1) were observed in both cohorts. Patients with delayed management were less likely to have a food bolus visualized (86.7% vs 63.3%; *P* = .025) and thus less likely to require endoscopic instrumentation. Accessory use was 24.4% and 20% (*P* = .781) in the early and delayed cohorts, respectively. Multivariable analysis showed no correlation among clinical demographics, presence of eosinophilic esophagitis, or procedural time with mucosal injury. Endoscopic instrumentation was associated with increased but not statistically significant odds of mucosal injury (odds ratio, 5.0; 95% confidence interval, 0.443-58.1; *P* = .192).

**Conclusions:**

Delaying endoscopic intervention beyond 6 hours of presentation does not result in significantly worse clinical outcomes. These results will help endoscopists risk stratify food bolus cases, possibly delaying intervention until daytime hours when more endoscopy personnel and resources are available.

## Introduction

Esophageal food bolus obstruction is a common reason for emergency department (ED) presentation, with an annual incidence of 13 out of 100,000 individuals.^1^ Although a large proportion will pass spontaneously into the stomach, 10% to 20% of cases will require urgent endoscopic intervention.^1^ They are often caused by underlying structural esophageal abnormalities, such as Schatzki rings, peptic strictures, and esophageal malignancies, as well as motility disorders such as achalasia.^2^^,^^3^ Eosinophilic esophagitis (EoE) is also quickly becoming a common cause of food bolus obstruction among younger patients, with a rising incidence of 5 to 10 cases per 100,000 individuals.^2^^,^^4^

Given the potential risk of adverse events such as aspiration, mucosal tearing, and perforation with prolonged obstruction, current guidelines recommend endoscopic intervention within 12 to 24 hours for complete soft food bolus obstruction of the esophagus.^5^ Previous reports have suggested endoscopic therapy as soon as 6 hours to decrease the likelihood of a piecemeal extraction.^6^ Current recommendations suggest first attempting to gently push the obstruction into the stomach.^5^^,^^7^ Endoscopic accessory use with a snare, basket, or overtube may be used if pushing alone is unsuccessful.^5^ Delaying intervention beyond 48 hours is not recommended given increased risk of perforation and surgical intervention.^5^ Severe adverse events such as critical care admission and death are rare, occurring in <1% of cases.^5^^,^^8^

Although emergency endoscopy is required for sharp objects or disk batteries, data regarding soft food bolus impactions are more limited.^1^^,^^5^ Retrospective evidence has suggested that delaying endoscopy after 12 hours from ingestion is not associated with an increase in morbidity or mortality.^8^, ^9^, ^10^ Recently, overall door-to-endoscopy time has not demonstrated any association with negative clinical outcomes or adverse events.^11^ In practice, many endoscopists prefer to intervene emergently, often within 2 to 6 hours after ingestion, as per the European Society for Gastrointestinal Endoscopy guidelines, to minimize risk of adverse events.^12^^,^^13^ Data regarding this more urgent timeframe remain lacking, to our knowledge.

Our study aimed to use real-world data to complement the existing sparse literature regarding the timing of endoscopic management for complete food bolus impactions. Specifically, we compare the rates of 30-day hospitalization and mortality, as well as mucosal tears, perforations, and need for endoscopic instrumentation between 2 cohorts who underwent emergent (within 6 hours) or delayed (after 6 hours) intervention.

## Methods

### Study design

We conducted a multicenter retrospective study involving 2 tertiary (Victoria General Hospital and Royal Jubilee Hospital [Victoria, British Columbia, Canada]) and 1 secondary care center (Sechelt General Hospital [Sechelt, British Columbia]). Ethics approval was obtained through the University of British Columbia Research Ethics Board. All patients presenting to the ED between January 1, 2019, and December 31, 2022, with an International Classification of Diseases 10th revision code for esophageal obstruction were reviewed. Each chart was then reviewed to ensure study eligibility. Inclusion criteria included patients 18 years or older referred for symptoms in keeping with a complete soft food bolus obstruction, as determined by clinical judgment of the referring emergency physician at the time of triage. All patients must have been referred to gastroenterology and completed an upper endoscopy during the same hospital visit. Patients who had nonfood/sharp foreign bodies, or those with partial/resolved food bolus impactions at the time of triage who were discharged home for outpatient endoscopy, were excluded. Patients who met the study criteria subsequently underwent retrospective electronic chart review for data abstraction by 2 clinician authors (T.H. and A.F.).

### Data collection and outcomes

Baseline clinicodemographics, including age, sex, proton pump inhibitor (PPI) use, and Charlson Comorbidity Index (CCI), were documented. Previous history of food impactions, dysphagia symptoms, and EoE were also noted, as well as dysphagia-to-triage and ingestion-to-endoscopy times. Primary outcomes included endoscopic adverse events such as mucosal injury, aspiration, and perforation, as well as mortality and hospitalization by 30 days (both all-cause and dysphagia-specific). Secondary outcomes include need for endoscopic instrumentation, accessory equipment use (snare, basket, piecemeal resection, and overtube use), sedation requirements (unsedated, conscious, and general anesthesia), and hospital length of stay. Other variables of interest included timing of esophagogastroduodenoscopy (daytime, evening, and overnight), concomitant esophageal pathology (EoE, mass, strictures, erosive esophagitis, and presbyesophagus), and removal success.

### Statistical analysis

Statistical analysis was performed using Statistical Package for the Social Sciences Statistics version 29 for MacIntosh (Apple Inc, Cupertino, Calif, USA). Categorical variables are expressed as proportions (n, %) and were analyzed using the Pearson χ^2^ or Fisher exact tests where applicable. Quantitative variables are expressed as means (standard deviation) and analyzed via Mann-Whitney U test. A multinomial logistic regression model was used to assess variables associated with mucosal injury. Covariates included door-to-endoscopy time, procedural length, endoscopic instrumentation, and optical features of EoE; these variables were predetermined to be potentially associated with endoscopic adverse events.^2^^,^^8^^,^^9^ Age, sex, CCI, and PPI use were controlled for, given potential confounding. *P* values <.05 were deemed significant.

## Results

### Study demographics

Of the 493 patients identified with potential food bolus impaction, 75 met the inclusion criteria and were selected for our study. Excluded patients are summarized in Supplementary Table 1 (available online at www.igiejournal.org). Mean triage-to-endoscopy time was 6.95 (5.4) hours. Patients were stratified into an emergent (endoscopy within 6 hours of triage, n = 45) and delayed (endoscopy after 6 hours of triage, n = 30). Baseline demographics such as age, sex, CCI, PPI use, personal history of EoE, and ingestion to triage times were similar between both groups (Table 1). As expected, patients in the emergent group had shorter ingestion-to-endoscopy times than the delayed group. The average door-to-endoscopy times were 3.44 (1.39) and 12.2 (4.87) hours for the emergent and delayed groups, respectively. All obstructions were comprised of meat or mixed food products. A greater proportion of patients in the delayed group had procedures performed during the daytime hours (37.8% vs 73.3%; *P* = .011) compared with the emergent group, which had a greater proportion of evening procedures (60% vs 16.7%; *P* = .001).

**Table 1:** Baseline demographics of patients who underwent urgent (<6 hours) or delayed (>6 hours) endoscopic intervention for esophageal food bolus obstruction

Variable	Door-to-endoscopy time		*P* value
	<6 hours (n=45)	>6 hours (n=30)	
Age (years)∗	58.8 (17.1)	55.1 (19.6)	.427
Male	30 (66.6)	20 (66.7)	1
Alcohol consumption	24 (53.3)	17 (56.7)	.816
Smoking	4 (8.9)	5 (16.7)	.470
No. of prescription medications∗	2.8 (3.2)	2.5 (2.9)	.846
Concurrent PPI usage	10 (22.2)	7 (23.3)	1
Charlson comorbidity index∗	2.16 (2.20)	2.27 (2.70)	.690
Previous dysphagia symptoms	36 (80)	22 (73.3)	.587
Previous food impaction	26 (57.8)	15 (50)	.637
EoE history	5 (11.1)	6 (20)	.330
Ingestion-to-triage time (hours)			.304
<6	22 (48.9)	19 (63.3)	.920
6-12	4 (8.9)	0	.717
12-18	9 (20)	4 (13.3)	.992
18-24	5 (11.1)	3 (10)	1
24-48	2 (4.4)	0	.920
>48	3 (6.7)	4 (13.3)	.963
Type of impaction			1
Meat	33 (73.3)	22 (73.3)	
Other	12 (26.7)	8 (26.7)	
Timing of EGD			<.001
8:00 AM-5:00 pm	17 (37.8)	22 (73.3)	.011
5:01 pm-11:59 pm	27 (60)	5 (16.7)	.001
12:00 am-7:59 am	1 (2.2)	3 (10)	.32
Ingestion-to-EGD time (hours)			<.001
<6	18 (40)	0	.0068
6-12	8 (17.8)	5 (16.7)	1
12-18	3 (6.67)	12 (40)	.0315
18-24	19 (42.2)	7 (23.3)	1
24-48	3 (6.67)	2 (6.7)	1
>48	3 (6.67)	4 (13.3)	.963
Sedation used			.352
Conscious	39 (86.7)	28 (93.3)	1
Deep	3 (6.67)	2 (6.67)	1
Awake	3 (6.67)	0	.497

*EGD*, Esophagogastroduodenoscopy; *EoE*, eosinophilic esophagitis; *PPI*, proton pump inhibitor.

∗ Data represented as n (%) or as mean (standard deviation).

### Primary outcomes

Primary outcomes are displayed in Table 2. No mortality was observed by 30 days after endoscopy in either group. Three patients in the emergent group required readmission within 30 days (6.7%), among which 2 were directly dysphagia-related (4.4%). One patient had diabetic gastroparesis and re-presented in 10 days with recurrent dysphagia. Endoscopy was not performed, and the patient was discharged uneventfully. The second patient was found to have an esophageal mass and was admitted 17 days later for ongoing dysphagia. In contrast, 4 patients in the delayed group presented again to the hospital within 30 days (13.3%) (*P* = .427); however, only 1 was dysphagia-related (3.3%) (*P* = 1). This patient had endoscopic evidence of EoE but returned in 12 days with recurrent symptoms because of PPI nonadherence. Only 2 patients in either cohort required hospitalization for longer than 24 hours. There were no reported cases of aspiration, surgical intervention, or critical care admission. Only 1 patient in the urgent cohort sustained an esophageal perforation, which was treated nonoperatively. This was secondary to significant intraprocedural retching leading to a presumed full-thickness esophageal tear. Although there was a numerical increase in the rate of mucosal injury in the delayed cohort, this was not statistically significant (1/45, 2.2% vs 4/30, 13.3%; *P* = .150). Procedural time was also similar between the 2 cohorts.

**Table 2:** Comparison of primary outcomes of 30-day mortality, rehospitalization, length of stay, and endoscopic adverse events in patients who underwent urgent versus delayed endoscopy for food bolus obstruction

Variable	Door-to-endoscopy time		*P* value
	<6 hours (n = 45)	>6 hours (n = 30)	
Mortality by 30 days	0	0	N/A
Readmission by 30 days			
All-cause	3 (6.7)	4 (13.3)	.427
Dysphagia-related	2 (4.4)∗	1 (3.3)†	1
Length of stay			1
<1 days	43 (95.6)	28 (93.3)	
1 days	2 (4.4)	2 (6.67)	
>1 days	0	0	N/A
Adverse events			
Aspiration	0	0	
Perforation	1 (2.2)	0	
Mucosal injury	1 (2.2)	4 (13.3)	.150
Surgical intervention	0	0	
Procedure time‡	15.1 (9.42)	15.7 (19.4)	.348

*N/A*, Not calculated because of lack of data.

∗ Ingestion-to-endoscopy time was 6 to 12 hours and >48 hours, respectively.

† Ingestion-to-endoscopy time was 18 to 24 hours.

‡ Data represented as n (%) or mean (standard deviation).

To interrogate for differences between time of ingestion-to-intervention, groups were restratified based on presentation within 12 hours (n = 31, “early”) or after 12 hours (n = 44, “prolonged”) from dysphagia onset to endoscopy. There was a numerical difference in rates of 30-day all-cause ED re-presentation of 1/31 (3.2%) versus 6/44 (13.6%) between the early or prolonged groups, respectively, that was not statistically significant (*P* = .229). However, dysphagia-related re-presentations were similar in the early (3.2%) versus prolonged groups (4.5%) (*P* = 1). Similarly, there was no significant difference (*P* = .397) in rates of mucosal injury (1/31 [3.2%] “early” versus 4/44 [9.1%] “prolonged”). The sole patient who presented with a perforation in our cohort had endoscopy performed within 6 hours of ingestion. Of the 4 patients who had mucosal injury in the delayed cohort, 2 had an endoscopy performed 12 to 18 hours after ingestion: 1 between 24 to 48 hours and another after 48 hours.

### Endoscopic instrumentation

Despite an initial clinical presentation with a complete food bolus obstruction, compared with those with early intervention, patients who underwent delayed endoscopy had a lower rate of visualized food bolus and thus need for endoscopic instrumentation (86.7% vs 63.3%, *P* = .025) (Table 3). The overall rate of spontaneous food bolus passage was 22.6% overall (13.3% early vs 36.6% delayed). Of the patients who required instrumentation, endoscopic accessory use was required in 24.4% and 20% of early versus delayed interventions, respectively (*P* = .781). Frequency of snare, basket, piecemeal, and overtube use is highlighted in Table 3, again showing no differences. Successful food bolus removal was achieved in all but 1 case in the early endoscopy group. This patient required hospital admission for a repeat endoscopy the following day, resulting in successful removal. In contrast, all patients in the delayed group had successful removal during the index endoscopy.

**Table 3:** Comparison of secondary outcomes of rates of visualized food bolus, endoscopic accessory use, and EoE in both patient cohorts

Variable	Door-to-endoscopy time		*P* value
	<6 hours (n = 45)	>6 hours (n = 30)	
Food bolus visualized	39 (86.7)	19 (63.3)	.025
Successful removal	44 (97.4)	30 (100)	1
Endoscopic removal technique			
Push	33 (73.3)	17 (56.7)	.144
Endoscopic accessory use	11 (24.4)	6 (20.0)	.781
Snare	8 (17.8)	4 (13.3)	.752
Basket	3 (6.7)	1 (3.3)	.646
Piecemeal	4 (8.9)	3 (10)	1
Overtube	2 (4.4)	0	.514
Positive endoscopic findings on scope	41 (91.1)	25 (83.3)	
EoE	20 (44.4)	14 (46.7)	1
Mass	2 (4.4)	0	.514
Stricture	23 (51.1)	13 (43.3)	.638
Barrett’s esophagus	0	0	
Erosive esophagitis	9 (20)	7 (23.3)	.778
Presbyesophagus	5 (11.1)	0	.079
Biopsy samples taken for EoE	19 (42.2)	13 (43.3)	1
Biopsy samples positive for EoE	10 (22.2)	9 (30)	.589

Data represented as n (%).

*EoE*, Eosinophilic esophagitis.

### Anesthesia requirements

No overall differences were observed in the anesthetic modalities used in each cohort (Table 2). Conscious sedation was the most used form of procedural anesthesia in both groups (86.7% emergent, 93.3% delayed; *P* = 1). General anesthesia was used in 6.67% of cases in both cohorts. Three patients in the early cohort elected to undergo endoscopy without sedation (6.67%).

### Endoscopic findings

Endoscopic findings of interest included EoE, mass, stricturing, erosive esophagitis, and presbyesophagus. The emergent cohort had 41 patients (91.1%) who had positive endoscopic findings, compared with 25 patients (83.3%) in the delayed cohort. Rates of individual findings are highlighted in Table 3, again showing no differences. Endoscopic features of EoE were identified in 20 patients (44.4%) and 14 patients (46.7%) in the emergent and delayed groups, respectively. This subsequently resulted in an incidence of 22.2% and 30% (*P* = .589) of biopsy-proven EoE in each respective group. No cases of Barrett’s esophagus resulting in food bolus impaction were observed in either group.

### Multivariable analysis

To assess for factors potentially associated with mucosal injury, a multivariable logistic regression was performed. Covariates of interest included age, CCI, procedural duration, door-to-endoscopy time, sex, endoscopic instrumentation, PPI use, and endoscopic features of EoE. Overall, there was no association between any covariate and mucosal injury (Table 4). Although endoscopic instrumentation resulted in an increased association with mucosal injury, this was not statistically significant (odds ratio, 5.0; 95% confidence interval, 0.443-58.1; *P* = .192).

**Table 4:** Multivariable analysis of clinicodemographic-adjusted odds of mucosal injury visualized on index endoscopy

Variable	Odds ratio (95% confidence interval)	*P* value
Age (years)	1.03 (0.932-1.140)	.561
Charlson comorbidity index	0.99 (0.528-1.86)	.974
Door-to-endoscopy time	0.998 (0.995-1.001)	.164
Procedural duration	1.03 (0.891-1.18)	.734
Endoscopic instrumentation	5.07 (0.443-58.1)	.192
Sex	0.276 (0.018-4.346)	.360
PPI use	0.252 (0.027-2.35)	.226

*PPI*, Proton pump inhibitor.

## Discussion

Complete esophageal soft food bolus impaction remains a common emergency in the gastroenterology field.^3^ Although previous evidence regarding timing of nonfood, sharp, or button battery ingestion is quite robust, data for soft food bolus impactions remain limited^1^^,^^5^^,^^12^ Here, we present outcomes after emergent (<6 hours) and delayed (>6 hours) endoscopic intervention, showing no significant difference in hospital readmission or mucosal injury in either group.

Our results build on a previous report by Krill et al,^8^ who similarly compared outcomes associated with “early” endoscopy within 12 hours versus “delayed” endoscopy after 12 hours of ingestion. In addition to ingestion-to-endoscopy time, here we report outcomes based on door-to-endoscopy time, which may better represent real-world triaging timelines regardless of times of dysphagia onset. We also included patients based on complete obstructive symptoms at presentation, as determined by the emergency physician, as opposed to symptoms at the time of intervention, to better reflect real-world endoscopic triaging. Although we did not have any aspiration events or mortality, we found a lower rate of mucosal tearing in our <6-hour group (2.2%) than the 16.7% in their “early” cohort. In contrast, our >6-hour group had a higher tear rate (13.3%) than their >12-hour cohort (5.9%). This aligns with findings from DiLeo et al,^14^ who found no adverse events occurred in patients who had endoscopy within 6 hours of symptom onset. Although the Krill et al^8^ study suggests a 12-hour cutoff may be safe, the European Society for Gastrointestinal Endoscopy guidelines recommend urgent endoscopy within 2 to 6 hours, which is more in keeping with real-world practices at our center.^13^ By assessing stratifying patients at a 6-hour cutoff, our data suggest that despite presentation of triage symptoms of complete obstruction, delaying endoscopy beyond 6 hours increases the chance of spontaneous passage without any increased risk of adverse events. However, our multivariable analysis did not identify a statistically significant association between door-to-endoscopy time and increased odds of mucosal injury in keeping with a recent report which did not identify any association between door-to-endoscopy time and adverse events.^11^ Our reported perforation rate of 2.2% in the emergent cohort was also in keeping with previous findings^8^^,^^11^^,^^14^

Krill et al^8^ also found that endoscopic accessory use resulted in increased rates of mucosal injury.^8^ In our multivariable analysis, which encompassed all endoscopic interventions, including the commonly used and effective “push” method, we did not identify correspondingly increased odds of mucosal injury.^6^^,^^7^ Rather, we found that patients in the delayed group had significantly lower odds of requiring endoscopic intervention. This was primarily because, with time, the food bolus was likely to pass into the stomach spontaneously. Although we report an overall spontaneous passage rate of 22.6%, others have reported this to be as high as 47.9%.^15^ This may suggest that delaying intervention beyond 6 hours may also decrease the need for overall endoscopic intervention, without evidence of significant adverse events. Given our emergent cohort had a higher rate of endoscopy after 5:00 pm, these data would also suggest that delaying intervention beyond 6 hours could also decrease the need for after-hours endoscopy with limited staffing. This is reflected in our data because the majority of “delayed” procedures occurred during daytime hours (having been held over from overnight). Most urgent endoscopies occurred during the evening, suggesting that cases requiring emergent intervention, as determined by physician judgment, were completed regardless of time of day.

### Aspiration/anesthesia

Aspiration is a worrisome adverse event of complete food bolus impaction, and the inability to clear secretions is a perceived risk factor that prompts more urgent endoscopy.^1^^,^^5^^,^^16^ We did not identify any aspiration events in either cohort, although previous reports have suggested the overall risk is low (1.5%-2.4%).^8^ To mitigate risks of aspiration, endotracheal intubation is often recommended for patients with challenging sedation requirements, high-grade obstructions, or expected prolonged procedures.^1^^,^^5^ We found a 6.67% rate of general anesthesia use in both our cohorts, which is significantly lower than previous reports and may be attributable to the fact that conscious sedation is generally preferred by endoscopists at our centers.^8^

### Endoscopic findings

With the increasing incidence of EoE in North America, we found a prevalence of 25.3% of biopsy-proven disease in our cohort (59.3% of patients with biopsy specimens taken). This is similar to what was reported by Krill et al,^8^ although previous prevalences during food bolus removal have ranged from 20% to 33%.^1^^,^^2^^,^^5^^,^^17^ Our EoE prevalence was second only to strictures as a cause for dysphagia, highlighting the importance of routine EoE sampling during food bolus endoscopies.

### Limitations

Our study has several limitations. The retrospective nature of our study prevented us from controlling the door-to-endoscopy time. Most complete food bolus obstructions performed at our center are typically done so on an emergent basis (within 6 hours), and thus, we did not have sufficient statistical power to comment on outcomes associated with longer door-to-endoscopy times. There is also a selection bias whereby patients in the delayed group may have been deemed clinically stable enough to wait per the physician judgment. We also report a lower rate of negative clinical outcomes relative to the number of procedures performed compared with similar studies. Thus, we could not comment on several outcomes, such as death or aspiration. This could again be attributable to the frequency of emergent endoscopy at our centers, with few patients delayed beyond 12 hours. Although we had access to the electronic medical records of all hospitals within the Vancouver Island Health Authority, patients presenting elsewhere with adverse events would have been missed. We also rely heavily on emergency physician judgment to operationally define a complete obstruction, as well as the patient's own reporting of time of ingestion and dysphagia onset. We could not account for patient self-reported discomfort while waiting for endoscopy, given the retrospective nature of our data, a significant clinical factor to account for while triaging.

## Conclusion

In conclusion, we show that delaying door-to-endoscopy time beyond 6 hours increases the chance of spontaneous food bolus passage without associated increased risk of hospital readmission or adverse events. Our findings align with growing retrospective data suggesting no increased negative clinical outcomes with delaying intervention for esophageal food bolus by up to 6 to 12 hours from time of ingestion. By filling this knowledge gap, we hope to assist endoscopists with risk stratification, potentially delaying procedures until a more convenient and fully staffed timeslot for safe endoscopy can be arranged.

## Patient consent

No patient identifiers were used in this study.

## Disclosure

All authors disclosed no financial relationships.

## Figures and Tables

**Supplementary Table 1:** Exclusion criteria

No. of patients (n = 418)	Reason for exclusion
217	Age <19
77	Nonfood obstructions
73	Resolution of esophageal obstruction/dysphagia symptoms
27	Bone/sharp food ingestions
14	Referred to otolaryngology/pulmonary for removal
10	Endoscopy not performed for unspecified reason
